# Gender differences and left-behind experiences in the relationship between gaming disorder, rumination and sleep quality among a sample of Chinese university students during the late stage of the COVID-19 pandemic

**DOI:** 10.3389/fpsyt.2023.1108016

**Published:** 2023-05-05

**Authors:** Li Li, Ligang Liu, Zhimin Niu, Huahua Zhong, Songli Mei, Mark D. Griffiths

**Affiliations:** ^1^School of Humanities and Social Sciences, Gannan Medical University, Ganzhou, China; ^2^School of Economics and Management, Jiangxi University of Science and Technology, Ganzhou, China; ^3^School of Public Health, Jilin University, Changchun, China; ^4^International Gaming Research Unit, Psychology Department, Nottingham Trent University, Nottingham, United Kingdom

**Keywords:** gaming disorder, rumination, sleep quality, left-behind experience, network analysis

## Abstract

**Background and aims:**

Studies have shown that gaming disorder (GD) is associated with rumination and poor sleep quality. However, the reciprocal relationship between GD, rumination and sleep quality is unclear. Moreover, the differences between gender and between left-behind experiences in the aforementioned relationship remain unknown. Therefore, the present study examined gender differences and left-behind experiences in the relationship between GD, rumination, and sleep quality among a sample of Chinese university students during the late stage of COVID-19 pandemic using a network analysis approach.

**Methods:**

A cross-sectional online survey of 1,872 Chinese university students was conducted comprising demographic information (age, gender, and left-behind experience), gaming experience, gaming frequency, Gaming Disorder Test (GDT), Short Version of Rumination Response Scale (RRS), and Pittsburgh Sleep Quality Index (PSQI).

**Results:**

Among Chinese university students, the prevalence of (i) GD was 3.5% and (ii) sleep disturbance was 14%. GD had positive and weak connection with rumination and sleep quality in the domain-level relational network. The network structures and global strengths both showed no significant differences between gender and between left-behind experiences. The nodes gd3 (*“continuation or escalation of gaming”*) and gd4 (*“gaming problems”*) had the strongest edge in the network.

**Conclusion:**

The results suggest reciprocal relationships between GD, rumination, and sleep quality. Gender and left-behind experiences did not influence the reciprocal relationship between GD, rumination, and sleep quality during the late stage of COVID-19 pandemic. Using network analysis, the findings provide novel insights that rumination and sleep quality may have interacted with GD among Chinese students during the late stage of COVID-19 pandemic. Reducing or eliminating negative rumination may decrease GD and improve sleep quality. Moreover, good sleep quality contributes to positive rumination which may decrease the risk of GD among Chinese university students.

## 1. Introduction

### 1.1. Gaming disorder

Gaming disorder (GD) was recently included in the category “disorders due to addictive behaviors” in the 11th revision of the International Classification of Diseases (ICD-11), and comprises both online and offline variants (1). In China, the Disease Prevention and Control Bureau of China reported that most gaming addicts prefer online gaming, such as Massively Multiplayer Online Role-Playing Games (MMORPGs) and Multiplayer Online Battle Arena (MOBA) games (2). Therefore, GD in the present study refers to GD predominantly online [i.e., internet gaming disorder (IGD)]. GD may compromise real-life interpersonal relationships and academic performance, erode family harmony and impair psychological and physical health, resulting in such consequences as anxiety, depression, eye strain, and wrist pain (3, 4). As of December 2021 in China (where the present study was carried out), the number of online gamers was more than 553 million (53.6% of Chinese internet users), an increase of nearly 70 million compared to before the novel coronavirus disease-2019 (COVID-19) outbreak (483 million online gamers in December, 2018) (5, 6).

The COVID-19 pandemic has disrupted global daily life in lots of aspects, including the consumption of gaming (7). During the COVID-19 outbreak, many countries advised or ordered their citizens to stay at home to prevent the spread of the virus. Individuals confined to their home turned to online gaming and other online activities as a source of entertainment or leisure. Some scholars have indicated that gaming activities may have conferred lots of benefits during the COVID-19 pandemic, such as social communication and coping with negative emotion and stress (8). However, other research has suggested that videogame playing and GD has increased significantly among adolescents and emerging adults during the COVID-19 pandemic (9, 10).

### 1.2. Rumination

Nolen-Hoeksema, Wisco and Lyubomirsky (p. 400) defined rumination as “the process of thinking perseveratively about one’s feelings and problems rather than in terms of the specific content of thoughts” (11). Rumination is associated with maladaptive cognition including unreasonable attribution, hopelessness, excessive self-criticism, and pessimism (12–14). Watkins and Roberts proposed a H-EX-A-GO-N model explaining the onset and maintenance of rumination (15). Habit development (H) and the perception of goal discrepancies (GO) lead to trait and state rumination, respectively. Abstract processing (A) and negative biases (N) may influence rumination, while executive functioning deficits (EX) may also cause depressive rumination (15). In addition, rumination and fear of COVID-19 were found to mediate the association between intolerance of uncertainty and mental well-being (16). Co-rumination following COVID-19 was regarded as a form of psychological inflexibility, which was associated with internalizing symptoms (e.g., passive attention and repetitively discussing problems) (17). Rumination may also exacerbate and prolong psychological distress and disturb sleep maintenance (11). In addition, a survey found that female adolescents reported more rumination and depression than males (18).

### 1.3. Sleep quality

Sleep quality is closely linked to individuals’ health in many circumstances. Some scholars have indicated that the COVID-19 pandemic may have increased sleep problems and damaged the immune system function (19). Lockdown and self-quarantine during the COVID-19 pandemic have also changed sleep schedules and deteriorated sleep quality including postponing bedtime and waking time, reducing sleep at night time, and increasing napping during the day time (20). Insomnia induced by stressful events is a common body reaction, especially in the face of the COVID-19 pandemic. A meta-analysis from seven studies reported that the prevalence of sleep disturbance was 23% among Chinese higher education students during the COVID-19 pandemic (95% CI: 14%–32%) (21). A report indicated that sleep quality among university students during COVID-19 was worse than that prior to the pandemic (22). Moreover, depression, anxiety, and poor psychological resilience are associated with poor sleep quality.

### 1.4. Gender differences

There are significant gender differences in many mental disorders (e.g., anxiety disorders, depressive disorders, eating disorders, and GD) (23–26). In addition, females may experience more rumination than males (27). Lee, McEnany and Weekes reported gender differences in sleep patterns including more daytime sleepiness and large variations between weekday and weekend sleep schedules among young male adolescents compared to female adolescents (28).

### 1.5. Left-behind experiences

Left-behind experiences refer to individuals in childhood who have experienced the situation of being left behind in a rural region of China at least 6 months under the care of kin members, while their parents became migrant workers in urban areas (29, 30). Left-behind children have been found to be more vulnerable to GD compared to non-left-behind children due to lack of parental care and supervision (31). In addition, left-behind experiences may influence an individual’s mental health including Chinese university students (32, 33). In relation to some of the aforementioned variables, Yang and Liu reported that life events, self-esteem, and rumination were all associated with internalization problems among children with left-behind experiences (34). Moreover, children with left-behind experiences have been found to show more sleep problems and have been found to exhibit a higher prevalence of sleep problems (35).

### 1.6. The present study

To date, the relationship between gaming disorder, rumination, and sleep quality during the COVID-19 pandemic has not been investigated, especially utilizing network analysis. In addition, gender differences and left-behind experiences in relation to gaming disorder, rumination, and sleep quality among Chinese university students during the late stage of COVID-19 pandemic are also unclear. Therefore, gender differences and left-behind experiences between the aforementioned variables among Chinese university students during the late stage of the COVID-19 pandemic need to be examined.

## 2. Theoretical background

Based on the Conservation of Resources Theory (CRT) (36), individuals are motivated to retain, protect, and build all kinds of resources for surviving and maintaining well-being (37). The COVID-19 pandemic has changed some individuals’ behavioral style, and impacted on their psychological and physical health (38). As for university students, they could not go to university and needed to take online classes at home, which may have made them anxious and depressed due to lack of face-to-face communication. One study surveying 405 Chinese university students reported that 44% had depression symptoms, 42.2% felt anxiety, and 29.4% had experienced stress because of home quarantine during the COVID-19 pandemic (39). Another study reported that 7.3% of Chinese college students had comorbidity of depression and anxiety because of home quarantine during the COVID-19 pandemic (40). Tang et al. reported more severe alexithymia among home-quarantined Chinese university students with depression (*n* = 223) or PTSD (*n* = 73) compared to home-quarantined Chinese university students without depression (*n* = 2,262) or PTSD (*n* = 2,412) during the pandemic (41). Finally, in a study among 7,800 Chinese college students, Ye et al. indicated the COVID-19 pandemic outbreak might induce acute stress disorder (ASD) due to home quarantine in the epidemic regions and found that the relationship between COVID-19-related stressful experiences (e.g., home quarantine) and ASD were mediated by resilience, better coping strategies, and social support (42).

For protecting and rebuilding various essential resources involving psychological and social resources (e.g., reducing stress and negative emotion due to COVID-19 and increasing social connection through online gaming), some individuals experiencing resource loss may try to escape from stress or increase social communication through engaging in problematic behaviors, such as substance use and gaming (37).

In the Interaction of Person-Affect-Cognition-Execution (I-PACE) model (43), predisposing (within-person) variables involving genetics, early childhood experiences (e.g., being left-behind), coping style (e.g., rumination), psychopathology, needs, motivation, and values, which arouse individuals’ perception of external stimulus (online gaming) and demonstrate affective and cognitive responses, increasing gaming behaviors, and perhaps leading to GD in a minority of cases. In addition, based on the Responses Styles Theory (RST) (44), negative rumination as a cognitive bias and coping style may lead to gaming cue-reactivity and craving, further causing or exacerbating GD, while GD may also increase the experience of rumination.

Sleep quality is closely associated with physical, psychological, and social environmental factors, such as insomnia due to a medication or psychoactive substances, poor cognition and/or stress (e.g., rumination, PTSD and COVID-19) (45). For relieving and escaping negative emotion or satisfying social communication need during the COVID-19 pandemic, some adolescents and emerging adults engage in persistent or recurrent gaming behaviors, and ignore negative outcomes, such as poor academic performance and sleep disturbance, which may intensify individuals’ reflection (i.e., rumination). Individuals may lose motivation to study hard and be trapped in self-denial anxiety based on the Self-Regulation Executive Function theory (S-REF) (46), which may impact sleep quality and further increase GD.

The power control theory has postulated that sex-role socialization may contribute to (i) taking risks and higher impulsivity among males and (ii) aversion and better self-control among females (47, 48). Gender rooted in social and cultural factors rather than biological, social, political, economic, and cultural differences and may produce different health risks between genders (49). In terms of the theory of children’s psychological development, parent–child communication is one of the most important factors in promoting better child development (50). However, for better job opportunities and higher salaries, lots of rural parents in China have to work away from their family and are unable to take care of or supervise their children (i.e., left-behind children) due to lack of face-to-face communication. Consequently, left-behind children may experience poor school achievement, as well as greater physical and psychological health problems (51). Therefore, the gender and left-behind experiences were considered as the moderated variables to influence the interaction of the variables in the present study (i.e., GD, sleep, and rumination).

The network analysis approach has been applied widely in many fields of scientific research over the past 20 years. In the field of psychiatric research, network analysis has helped to explain mental disorders’ core symptoms, comorbidity issues, the interaction of symptomatic elements, and possible influencing factors (52). Using network analysis, Yuan et al. found that the core symptoms of GD included preoccupation, loss of control (53), and gaming irrespective of negative outcomes using the nine-item Internet Gaming Disorder Questionnaire (54). Collins et al. identified three components of rumination including brooding, reflective pondering, and difficulty trusting positive feelings using a network analysis approach (55). In addition, Marques and de Azevedo proposed the potentialities of network approach for sleep medicine, such as accounting for the psychological structure and relationship between sleep problems, and describing comorbidity conditions (56).

In the motivation-cognition-behavior model of Internet Gaming Disorder (IGD), rumination as a maladaptive cognition is a high-risk factor of IGD (57). Moreover, GD may increase rumination about gaming behaviors and result in a vicious cycle of GD (57). Some studies have reported that the severity of GD is associated with poor sleep quality and greater psychological distress among Hong Kong university students (58). GD may cause negative consequences including sleep disturbance, poor academic performance, and psychological distress (45). In addition, sleep problems may also cause or exacerbate various mental disorders including internet addiction and GD (59, 60). Rumination has been found to be positively associated with subjective sleep quality after controlling variables of negative mood (61). Zoccola, Dickerson, and Lam also indicated that rumination may predict longer sleep onset latency after individuals experience acute psychological stress (62). In addition, the inability to fall asleep after going to bed may trigger ruminative thought (63). Qiu et al. have indicated that online risky behavior including GD and social networking site addiction may impact on sleep quality through rumination and anxiety as mediators among Chinese university students (64). Based on the dysfunctional metacognitions concerning gaming (65), individuals with IGD often tend to ruminate (i.e., thinking out videogames) even when they are not gaming, which may increase sleep disturbance and lead to a vicious cycle (57).

## 3. Hypotheses

Most studies have indicated that males have higher prevalence of GD than females (26, 66, 67). Some scholars have also indicated gender differences in rumination with females being more likely than males to engage in rumination (27, 68). In addition, female students have been found going to bed and getting up earlier, having longer sleep latency, and poorer sleep quality than males (69). Based on the aforementioned literature, it was hypothesized that there would be gender differences in network structure and global strength of GD and rumination and sleep quality (H_1_).

Left-behind experiences are predictive of a greater risk of psychiatric morbidity for university students into early adulthood (70). Individuals with left-behind experiences are more likely to be at higher risk of behavioral addiction (e.g., GD and internet addiction) (31). Left-behind experiences are also associated with rumination and poor sleep quality, respectively (34, 35). Based on the aforementioned literature, it was hypothesized that the network structure and global strength of GD and rumination and sleep quality will be different between those with left-behind experiences and those without left-behind experiences (H_2_).

## 4. Methods

### 4.1. Participants and procedure

The present study was a cross-sectional survey investigation. Convenience sampling was utilized to collect data from eight universities in four provinces of China (i.e., Heilongjiang, Jiangxi, Liaoning, and Shannxi) from September, 2021 to December, 2021. Participants were recruited with the incentive of gaining course credits. The total of 2,322 participants (Heilongjiang 456, Jiangxi 883, Liaoning 637, and Shannxi 346) completed the online survey. The inclusion criterion for the participants was to have played videogames in the past year. In addition, 440 non-gamers were excluded along with 10 participants due to missing data. Therefore, the remaining sample was 1872 Chinese university students (males = 930, females = 942) ranging from 17 to 24 years (mean = 19 years; SD = 1.7). Participants were informed of the study purpose and completed the survey voluntarily. The whole survey took approximately 10 min for participants to complete.

### 4.2. Measures

#### 4.2.1. Gaming experience, gaming frequency, and left-behind experience

Gaming experience was assessed with the question “How many years have you played videogames?,” and gaming frequency was assessed with the questions “How many days a week do you play videogames?,” and “How many hours do you play videogames on weekdays and weekends, respectively?.” Left-behind experiences was assessed by the question “Have you experienced the situation of being left behind in a rural region for at least 6 months, while your parent or parents became migrant workers in urban areas?” and answered either “Yes” or “No.”

#### 4.2.2. Gaming disorder

The four-item Gaming Disorder Test (GDT) was used to assess the severity of GD, and has been shown to have very good reliability and validity among Chinese university students (71). Items are rated on a five-point scale (*“never,” “rarely,” “sometimes,” “often,”* or *“very often”*). Pontes et al. recommended that at least one of items should be answered 4 (“often”) or 5 (“very often”) to distinguish between potentially disordered and non-disordered gamers (71). In the present study, the Cronbach’s alpha and McDonald’s ω were 0.833 (CI: 0.821~0.845) and 0.838 (CI: 0.825~0.850).

#### 4.2.3. Rumination

The 10-item short version of Rumination Response Scale (RRS) was used to assess the level of rumination and comprises two factors (i.e., brooding and reflection) (72). The RRS has been shown to have very good reliability and validity among Chinese university students (73). Every item is rated on a four-point scale from 1 (*“never”*) to 4 (*“very often”*), with higher scores indicating a higher level of rumination. In the present study, the Cronbach’s alpha and McDonald’s ω were 0.915 (CI: 0.909~0.920) and 0.916 (CI: 0.911~0.922).

#### 4.2.4. Sleep quality

The 19-item Pittsburgh Sleep Quality Index (PSQI) (74) was used to assess sleep quality. The PQSI has been shown to have very good reliability and validity among Chinese populations (75, 76). The PSQI comprises seven factors including subjective sleep quality, sleep latency, sleep duration, habitual sleep efficiency, sleep disturbance, used sleep medication, and daytime dysfunction. The PSQI global score ranges from 0 to 21 and a score of more than 7 indicates higher sleep disturbance symptoms or poorer sleep quality (74, 75). In the present study, the Cronbach’s alpha and McDonald’s ω were 0.646 (CI: 0.623~0.668) and 0.685 (CI: 0.663~0.707).

### 4.3. Statistical analysis

JASP software (77) was utilized to conduct statistical descriptions including demographic data, gaming experience and frequency, and the prevalence rates of GD and sleep disturbance. Other statistics included *t*-tests (i.e., gender differences and left-behind experiences), Pearson/Bayesian correlation analysis between variables, reliability coefficients of all scales, and EBICglasso network analysis. Cohen’s *d* and Bayesian correlation may provide effect sizes of significant difference. RStudio 3.4.4 software including qgraph and bootnet packages were used to calculate bridge centrality (78), edge-weight accuracy, centrality stability, and to test for significant differences (79), while NetworkComparisonTest packages were used to conduct network comparisons (80).

The network model was estimated to show the characteristics of node and edge as the important study variables through the graphic least absolute shrinkage and selection operator (LASSO) method, which is based on the Extended Bayesian Information Criterion (i.e., EBICglasso). The centrality of nodes was calculated through betweenness, closeness, strength and expected influence (81, 82). Bridge centrality was calculated through betweenness, closeness, strength, expected influence (1-step), and expected influence (2-step) to identify bridge symptoms (78), which may display the bridge symptoms between mental health problems. Edge-weight accuracy, centrality stability and testing for significant differences of nodes and edges need to be examined for assessing the network accuracy (79). The non-parametric bootstrap (i.e., 1,000 samples) was utilized to calculate edge-weight accuracy and testing for significant differences of nodes and edges, while the case-dropping subset bootstrap (95% confidence intervals) was utilized to assess the stability of centrality indices (79). The correlation stability coefficient (*CS*-coefficient, at least ≥0.25) indicated the node centrality stability (79). The stronger connection of nodes was indicated by thicker edge. The network comparison test (NCT) was carried out for gender and different left-behind experiences.

### 4.4. Ethics

The study was approved by the first author’s university Ethics Committee (Ref: 20BY184). Informed consent was provided by all participants.

## 5. Results

### 5.1. Descriptive statistics and correlation analysis

For GD, 632 participants (33.8%, males = 251, females = 381) chose “never” on all items, 1,174 participants (62.7%, males = 636, females = 538) reported “rarely” or “sometimes” for one item, 39 participants (2.1%, males = 26, females = 13) reported one indicator of GD (i.e., “often” or “very often”), 14 participants (0.7%, males = 9, females = 5) reported two indicators of GD, three participants (0.2%, males = 2, females = 1) reported three indicators of GD, and 10 participants (0.5%, males = 6, females = 4) reported all four indicators of GD (Appendix S1). The prevalence of GD (i.e., at least one indicator on the GDT) was 3.5%. In addition, 1,610 participants (86.0%, males = 801, females = 809) had good sleep quality, while 262 (14.0%, males = 129, females = 133) reported poor sleep quality. The mean number of hours of playing videogames on weekdays and weekends were 2.02 h (SD = 2.31) and 2.71 h (SD = 2.46), respectively. Of all participants, 616 university students had left-behind experience and 1,256 had no left-behind experience. In addition, mean value of rumination was 19.86 out of 40 (SD = 6.01).

The participants’ characteristics and the bivariate correlation analysis are presented in Tables 1, and 2, respectively. There were significant differences in GD, gaming experience (number of years spent gaming) and gaming frequency (hours spent gaming daily, number of days a week spent gaming) between gender (all *p*-values < 0.001, Cohen’s *d* > 0.4). There were no significant differences in GD, rumination, and sleep quality in relation to left-behind experiences (all Cohen’s *d* < 0.2). GD was significantly and positively associated with rumination, PSQI total score, subjective sleep quality, sleep latency, sleep disturbance, and daytime dysfunction (all *p*-values < 0.01, log[BF10] > 3). The frequencies of the seven PSQI factors are shown in Appendix S2. Using sleep medication had the lowest frequency (0.2%), while daytime dysfunction had the highest frequency (9%).

**Table 1:** Sociodemographic characteristics.

Variables	Total (*n* = 1,872)	Gender		*t*	*p*	*Cohen’s d*	Left-behind experience		*t*	*p*	*Cohen’s d*
		Male (*n* = 930)	Female (*n* = 942)				Yes (*n* = 616)	No (*n* = 1,256)			
Age (years)	19.0 ± 1.8	19.0 ± 1.8	19.0 ± 1.7	0.011	0.991	5.271e-4	19.22 ± 1.79	18.88 ± 1.72	3.997	<0.001	0.197
Years spent gaming	5.504.03	6.68 ± 4.06	4.34 ± 3.65	12.926	<0.001	0.607	5.36 ± 3.88	5.58 ± 4.10	1.105	0.269	0.055
Gaming days per week	3.65 ± 2.39	4.43 ± 2.29	2.88 ± 2.23	14.476	<0.001	0.684	3.72 ± 2.41	3.62 ± 2.38	0.809	0.418	0.041
Weekday gaming hours	2.02 ± 2.31	2.48 ± 2.64	1.56 ± 1.84	8.618	<0.001	0.406	2.08 ± 2.27	1.99 ± 2.34	0.777	0.437	0.039
Weekend gaming hours	2.71 ± 2.46	3.48 ± 2.87	1.95 ± 1.65	14.236	<0.001	0.658	2.82 ± 2.32	2.65 ± 2.52	1.369	0.171	0.067
Gaming disorders	6.45 ± 2.60	6.99 ± 2.78	5.93 ± 2.28	9.019	<0.001	0.417	6.73 ± 2.71	6.32 ± 2.53	3.201	0.001	0.157
Rumination	19.86 ± 6.08	20.07 ± 6.25	19.65 ± 5.91	1.472	0.141	0.068	20.12 ± 6.17	19.73 ± 6.03	1.309	0.191	0.064
Sleep quality	4.70 ± 2.67	4.55 ± 2.75	4.84 ± 2.58	2.405	0.016	0.111	5.02 ± 2.66	4.54 ± 2.66	3.619	<0.001	0.178

**Table 2:** Correlation analysis of the study variables.

	GD	Rumination	PSQI	SSQ	SL	SD	HSE	SDD	USM
Rumination	0.192^**^								
log(BF_10_)	31.687								
PSQI	0.235^**^	0.25^**^							
log(BF_10_)	49.613	56.529							
SSQ	0.216^**^	0.148^**^	0.713^**^						
log(BF_10_)	41.052	17.107	660.903						
SL	0.147^**^	0.137^**^	0.687^**^	0.482^**^					
log(BF_10_)	16.755	14.097	593.622	243.489					
SD	0.071^**^	0.114^**^	0.457^**^	0.154^**^	0.101^**^				
log(BF_10_)	1.200	8.732	215.499	18.974	6.071				
HSE	0.021	0.037	0.413^**^	0.118^**^	0.122^**^	0.286^**^			
log(BF_10_)	−3.114	−2.257	170.725	9.490	10.380	76.190			
SDD	0.170^**^	0.209^**^	0.629^**^	0.415^**^	0.389^**^	0.121^**^	0.054^*^		
log(BF_10_)	23.849	38.280	467.038	172.646	149.761	10.212	0.773		
USM	0.072^**^	0.071^**^	0.251^**^	0.123^**^	0.145^**^	0.060^**^	0.046^*^	0.105^**^	
log(BF_10_)	1.282	1.155	57.047	10.609	16.195	−0.145	−1.598	6.721	
DD	0.207^**^	0.244^**^	0.715^**^	0.433^*^	0.333^**^	0.208^**^	0.025	0.421^**^	0.099^**^
log(BF_10_)	37.306	53.945	665.008	190.590	106.537	37.708	−2.966	178.982	5.712

^**^p < 0.01. GD, Gaming disorder; PSQI, sleep quality; SSQ, Subjective sleep quality; SL, Sleep latency; SD, Sleep duration; HSE, Habitual sleep efficiency; SDD, Sleep disturbance; USM, Used sleep medication; DD, Daytime dysfunction.

### 5.2. EBICglasso network analysis

**Figure 1:**
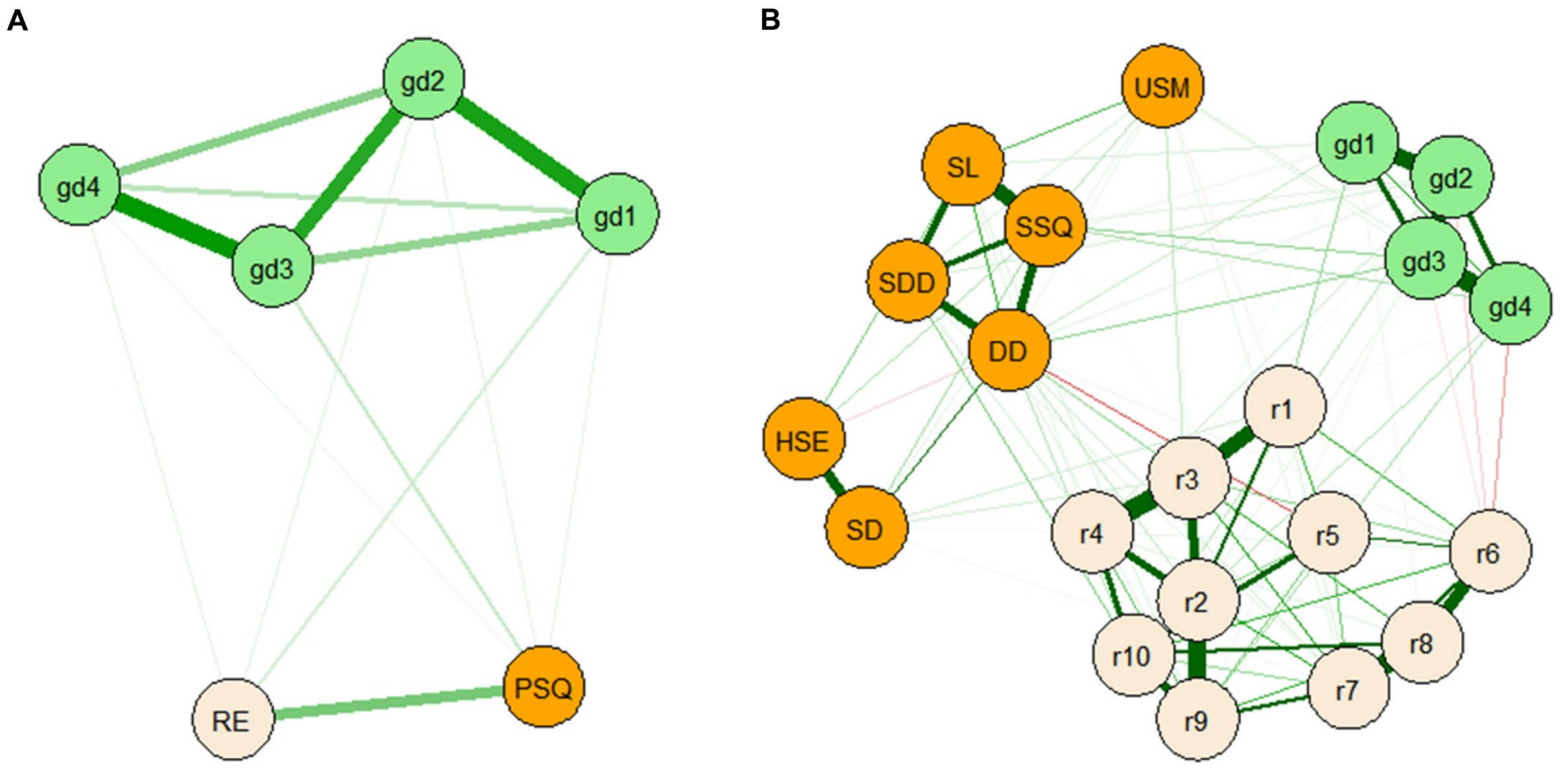
EBICglasso model based on the domain-level **(A)** and the item-level **(B)** network analysis according to the relationships between GD, rumination and sleep quality among 1,872 participants. gd1~gd4, Gaming disorder; RE, r1~r10 = Rumination; PQSI, Sleep quality; SSQ, Subjective sleep quality; SL, Sleep latency; SD, Sleep duration; HSE, Habitual sleep efficiency; SDD, Sleep disturbance; USM, Used sleep medication; DD, Daytime dysfunction.

**Figure 2:**
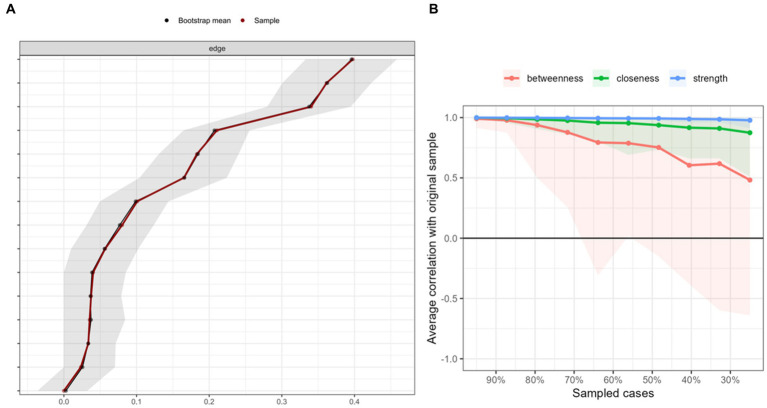
Bootstrapped confidence intervals of estimated edge-weights **(A)** and Case-dropping bootstrap procedure for node strength **(B)**.

**Figure 3:**
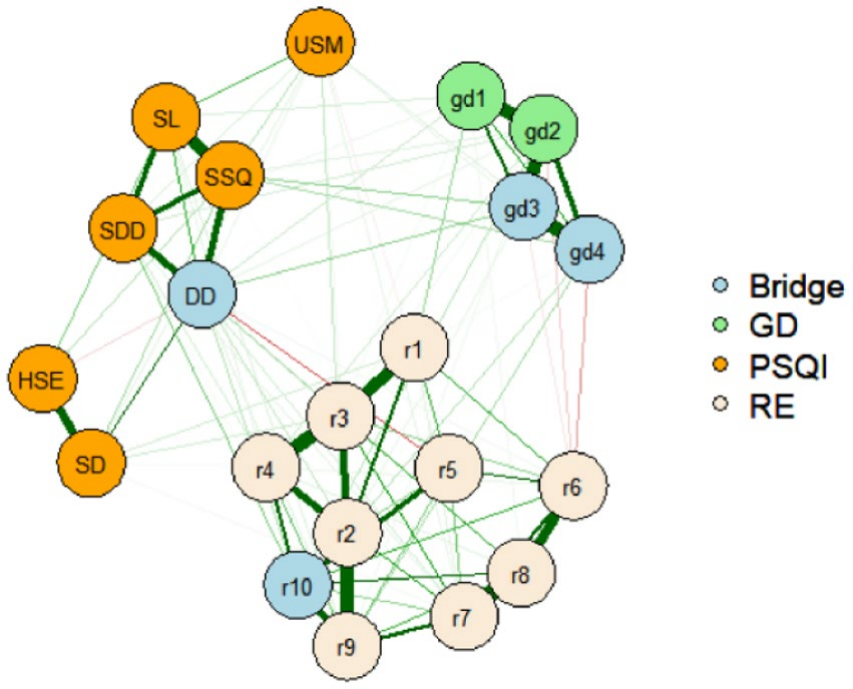
The item-level Network structure according to the relationships between GD, rumination and sleep quality among 1,872 participants. gd1~gd4, Gaming disorder; r1~r10 = Rumination; SSQ, Subjective sleep quality; SL, Sleep latency; SD, Sleep duration; HSE, Habitual sleep efficiency; SDD, Sleep disturbance; USM, Used sleep medication; DD, Daytime dysfunction. Nodes identified as bridge symptoms are colored in blue.

The GD, rumination, and sleep quality networks are shown in Figure 1A (i.e., the domain-level including four items of GD, and the total rumination and PSQI scores) and Figure 1B (i.e., the item-levels including the four GD items, the 10 rumination items, and the seven PSQI factors). GD was connected with rumination and sleep quality. In the 1A network, the strongest edge identified was between node gd3 (*“continuation or escalation of gaming”*) and gd4 (*“gaming problems”*; *r* = 0.476; Appendix S3), and gd3 was the strongest central node (betweenness = 1.379, closeness = 1.327, strength = 1.128, expected influence = 1.128; Appendix S4). The edge-weight accuracy and centrality stability are shown in Figures 2A,B, respectively. Most of edge-weights with wide bootstrapped CIs (Figure 2A) indicated that the order of the edges should be interpreted carefully. The CS-coefficient indicated that the node strength performed better [*CS*_(cor = 0.7)_ = 0.75; CS > 0.5]. The tests for significant differences are shown in Appendix S5. The edges gd3–gd4, gd1 (*“impaired control”*)-gd2 (*“increasing priority”*), and gd2–gd3 were significantly different from one another. All node strengths were significantly different from one another. In the 1B network, gd3 (*“continuation or escalation of gaming”*) and gd4 (*“gaming problems”*) had the strongest edge (*r* = 0.455). Moreover, sleep duration and habitual sleep efficiency (*r* = 0.436), and r3 (*“Think ‘Why do I always react this way?’”*) and r4 (*“Go away by yourself and think about why you feel this way”*; *r* = 0.424) also had stronger edges (Appendix S6). The node r3 had the highest strength and expected influence (Appendix S7). The CS-coefficient indicated that the node strength performed better [*CS*_(cor = 0.7)_ = 0.75; CS > 0.5]. The edge-weight accuracy, centrality stability, and tests for significant differences are shown in Appendix S8. In addition, the nodes gd3 (*“continuation or escalation of gaming”*), gd4 (*“gaming problems”*), r10 (*“go someplace alone to think about your feelings”*), and DD (*“daytime dysfunction”*) were significant bridge symptoms among those with GD (Figure 3). Bridge centrality indices are shown in Appendix S9. The bridge expected influence was 0.595 (0.517~0.672) when the maximum drop proportions retained a correlation of 0.7 in at least 95% of the samples.

### 5.3. Network analysis for gender and left-behind experiences

EBICglasso models based on the domain and item level network analysis between variables for gender and different left-behind experiences are shown in Appendix S10. In the domain-level network, the strongest edges identified were between node gd3 and gd4 among males (*r* = 0.454) and among females (*r* = 0.498; Appendix S11), and gd3 was the strongest central node (for males 1.355 and females 1.078; Appendices S12, S13). In the item-level network, r3 was the strongest node among males (2.566), while gd4 as the strongest node among females (1.40; Appendices S14, S15). In addition, for different left-behind experiences, nodes gd3 and gd4 both had the highest edge weight (for non-left-behind experience 0.492 and left-behind experience 0.424) in the domain-level network. Nodes gd3 and gd2 were the strongest central node among non-left-behind experience (1.166) and left-behind experience (1.010), respectively. Moreover, nodes r3 and used sleep medication (USM) were the strongest nodes among non-left-behind experiences (1.739) and left-behind experiences (3.596) in the item-level network, respectively (Appendices S16–S20).

### 5.4. Network comparison between gender

As for the NCT, the network structures and global strengths both had non-significant differences between gender at the domain-level (M = 0.085, *p* = 0.71; 2.09 vs. 1.99, *p* = 0.19) and the item-level (M = 0.140, *p* = 0.202; 8.47 vs. 7.88, *p* = 0.095), respectively.

### 5.5. Network comparison between different left-behind experiences

The network structures and global strengths also had non-significant differences between different left-behind experiences at the domain-level (M = 0.111, *p* = 0.38; 2.10 vs. 2.02, *p* = 0.351) and the item-level (M = 0.080, *p* = 0.998; 8.27 vs. 8.13, *p* = 0.777), respectively.

## 6. Discussion

In the present study, the prevalence of GD was 3.5% among Chinese university students based on Pontes et al.’s recommendation that at least one indicator of GD in the GDT was scored “often” or “very often” (to distinguish between disordered and non-disordered gamers) (71). This rate is similar to previous studies [3.3%, Kim et al. (83); 3.05%, Stevens et al. (26)]. The below-average rate (5%) (2) may be due to lack of a more representative sample of young people (e.g., the present study did not include adolescents at middle schools and high schools, vocational schools, and school-leavers). The prevalence of sleep disturbance was 14.0% among Chinese university students during the late stage of COVID-19 pandemic, which was consistent with a previous study (13.93% [PSQI ≥8]) (84). Zhou et al. reported that the prevalence of sleep quality was 18.4% using the same instrument and evaluation criteria (i.e., PSQI >7/≥8) among frontline health professionals during the COVID-19 outbreak (85).

Some studies have indicated poorer sleep quality due to using different assessment tools or the same assessment tool with different cutoff criteria (e.g., PSQI ≥ 5) (86, 87). Most Chinese university students in the present study reported good sleep quality which may be due to when the survey took place. At that time of data collection, there were very few cases of COVID-19 in mainland China and most of those reported were imported cases (total 25 cases of which 20 were imported cases) (88). Therefore, students are likely to have exhibited less sleep disturbance during the late stage of the COVID-19 pandemic compared to earlier stages.

Males were found to be gaming more frequently and at higher risk of GD than females in the present study, and is consistent with the findings from recent meta-analytic studies (26, 66). Males often show internalization problems and adopt maladaptive coping strategies owing to social isolation, achievement, and competition (67, 89), which may increase the risk of GD by escaping negative emotions or satisfying psychological needs. In addition, there were no significant differences in GD, rumination and sleep quality between participants who had left-behind experiences and those who did not (all Cohen’s *d* < 0.2). A previous study found that relative deprivation among children with left-behind experiences may predict GD, and that the relationship between relative deprivation and GD was mediated through deviant peer affiliation (90). Therefore, the impact of left-behind experiences on the variables studied here need to be further examined.

Correlation analysis between GD, rumination and sleep quality was consistent with previous findings which reported online risky behavior (e.g., excessive gaming) was positively associated with rumination and sleep quality, and indirectly impacted on sleep quality through the mediation of rumination and anxiety (64).

In the present study, the gd3 node (*“continuation or escalation of gaming”*) and gd4 node (*“gaming problems”*) had the strongest edge, which indicated that gd3 and gd4 may be considered as the core symptoms of GD diagnosis for psychologists and clinical psychiatrists. In addition, the gd3 node was positively connected with sleep quality, especially subjective sleep quality (SSQ) and daytime dysfunction (DD). The most plausible explanation is that problematic gamers spend so much time playing videogames that they sleep much less and experience poor sleep quality. In addition, r3 (*“Think ‘Why do I always react this way?’”*) and daytime dysfunction (DD) were the most important nodes in rumination and sleep quality, respectively. The “used sleep medication” (USM) factor connected with GD and rumination in the item-level network. Of all participants, only 2.4% used sleep medication at least once or more a week. The USM factor has been found to have a weaker correlation with the total PSQI than other factors among Chinese individuals without insomnia (75, 76). Some studies have found that online risky behaviors and internet addiction are associated with sleep quality, but indirectly rather than directly influenced sleep quality through multiple mediation (e.g., rumination and anxiety as mediating factors) (64, 91). Therefore, further research examining the relationship between GD and sleep quality is needed.

In addition, gd4 was connected negatively with r6 (“*Think about a recent situation, wishing it had gone better*”) and positively with r9 (“*Analyze your personality to try to understand why you are depressed*”), which indicates the close relationship between rumination and functional impairments of GD. In addition, the nodes gd3 (“*continuation or escalation of gaming*”), gd4 (“*gaming problems*”), r10 (“*go someplace alone to think about your feelings*”), and DD (“*daytime dysfunction*”) were significant bridge symptoms among those with GD. This indicates the close association between GD, rumination, and sleep. Rumination accompanied by going someplace alone may increase daytime dysfunction, and lead to GD for a minority of gamers.

In the I-PACE model (43), rumination as a negative coping style (i.e., one of general predisposing variables) may lead to GD and sleep disturbance, while GD may increase positive rumination (e.g., getting gaming rewards and complete gaming tasks) and negative rumination (e.g., poor interpersonal relationship and academic performance), further increasing sleep disturbance. Sleep problems with day and night reversal may also increase rumination and exacerbate GD (60). Wood, Griffiths, and Parke reported that the negative aspects of time loss included loss sleep and a guilt feeling of “wasting time” (i.e., rumination) among videogame players, which also suggests an interaction between GD, rumination, and sleep quality (92). GD, rumination, and sleep quality appear to be connected using the network analysis approach in the present study and appear to display reciprocal interactions between the three variables, which was verified by the analyses of network accuracy and stability.

In the present study, no gender differences in the network structures and global strengths were found. Therefore, H_1_ was not supported. Gender differences in GD may be associated with gaming motives and preferences, such as females liking gaming for building intimacy and recreation with males liking gaming for competition and escaping negative emotion (93, 94). More specifically, males prefer Massively Multiplayer Online Role-Playing Games MMORPGs and Multiplayer Online Battle Arena (MOBA) games, while females prefer casual games (95, 96). In addition, females have been found to experience greater rumination than males (26), which is inconsistent with the findings of the present study. This may be due to the different sampled populations (i.e., the present study used purely university students whereas the findings reported by Johnson and Whisman are from a meta-analysis with many different sampled populations) (27). More generally, rumination has been found to significantly predict online gaming addiction among Chinese adolescents (97). Moreover, gender has been shown to moderate the relationship between rumination and problematic online gaming, especially for males (98). Some studies from Europe noted that reports of country-specific COVID-19 deaths decreased subjective sleep quality, which predicted greater rumination and somatic complaints (99). Therefore, a longitudinal study examining the relationship between GD, rumination, and sleep quality should be conducted, especially to examine if the COVID-19 pandemic has had a sustained and strong psychological and physical impact on university students.

The numbers of left-behind children and migrant workers are increasing with high-speed economic development in China. Left-behind children report more psychopathology and less pro-social behaviors than non-left-behind children (100), while university students with left-behind experiences also have more mental health problems, such as somatization, depression, and anxiety (33). Therefore, the influence of left-behind experiences to the interaction of the aforementioned variables was examined based on the theory of children’ psychological development. However, differences in left-behind experiences in network structures and global strengths were not found in the present study, and did not support H_2_. The results indicated a similar network relationship between different left-behind experiences during the late stage of COVID-19 pandemic. Liu and Wang indicated that university students who had left-behind experiences during primary school had more mental health problems than those who had left-behind experiences at middle school (101), while being left-behind by two migrant parents was worse for mental health problems than being left-behind by only one migrant parent. These studies indicated that more complex factors (e.g., time of being left behind and being a single parent) may influence the relationship between left-behind experience and mental health (e.g., negative emotion and coping style), which may increase or decrease GD.

As of 25 November 2021, 2464.33 million doses of COVID-19 vaccine had been administered in China (102). Moreover, the majority of citizens have now had a third COVID-19 vaccine in mainland China. In addition, the government took strict measures to control the spread of COVID-19 coming into China (e.g., cutting off international flights and introducing quarantine measures for individuals entering mainland China from abroad), which provided health protection for Chinese citizens to live in greater peace and contentment. Therefore, during the late stage of COVID-19 pandemic, nearly all Chinese university students had conventional offline classes without quarantine and self-isolation when they took part in the survey. Apart from traveling abroad, life has now gone back to normal in China.

Some limitations need to be considered in the present study. First, the cross-sectional design and convenience sampling (eight universities in four provinces) meant the sample was not representative of all Chinese students and causal relationships between variables could not be determined. Second, sleep quality was not examined using objective and sophisticated experimental equipment but relied on subjective self-report. Self-report more generally is subject to other methods biases. Third, gamers were only defined based on the question *“Have you played video games in the past year?”* (“Yes” or “No”), which did not take into account individual variations in the frequency of gaming which could have impacted on the findings. Fourth, very little information was collected regarding gaming habits. Therefore, the validity of the variables concerning gaming experience and gaming frequency are somewhat restricted. Fifth, the types of videogames played were not examined, which may have limited the correlational analysis that could be conducted with various study variables (e.g., gender). In addition, the gaming genre engaged in and characteristics of left-behind experiences need to be further analyzed, such as investigating the number of parents who migrated to work elsewhere (i.e., mother, father or both), students’ age when left-behind, and students’ personality and resilience, all of which may have influenced mental health of students with left-behind experiences, as well as impacting GD, rumination, and sleep quality. Therefore, representative and objective assessment, as well as a trajectory analysis needs to be included in future studies.

## 7. Conclusion

The results suggested that GD was significantly associated with rumination and sleep quality. There were no significant differences found between gender and left-behind experiences in relation to GD in the network structures and global strengths during the late stage of COVID-19 pandemic. The findings provide novel insights that rumination and sleep quality may have interacted with GD among Chinese students during the late stage of COVID-19 pandemic. Reducing or eliminating negative rumination may decrease GD and improve sleep quality. Moreover, good sleep quality contributes to positive rumination, which may then decrease the risk of GD among Chinese university students.

## Data availability statement

The original contributions presented in the study are included in the article/Supplementary material, further inquiries can be directed to the corresponding authors.

## Ethics statement

The studies involving human participants were reviewed and approved by Gannan Medical university Ethics Committee (Ref: 20BY184). The patients/participants provided their written informed consent to participate in this study.

## Author contributions

LLi and LLiu conceived and designed the study. LLi and ZN performed the experiments and wrote the first draft of the paper. LLi and MG analyzed and interpreted the data. SM and HZ contributed reagents/materials/analysis tools. MG edited and contributed to the revised paper. All authors contributed to the article and approved the submitted version.

## Funding

This work was supported by China Scholarship Council Project 202008360125, Jiangxi University Humanities and Social Science Research Project XL20104, The Science Education Program Project “Thirteenth Five-Year Plan” of Jiangxi Province 2020GX184, Key project of Gannan Medical University ZD201838, and Research and Innovation Team of Gannan Medical University TD2021RW01.

## Conflict of interest

MDG has received research funding from Norsk Tipping (the gambling operator owned by the Norwegian government). MDG has received funding for a number of research projects in the area of gambling education for young people, social responsibility in gambling and gambling treatment from Gamble Aware (formerly the Responsibility in Gambling Trust), a charitable body which funds its research program based on donations from the gambling industry. MDG undertakes consultancy for various gambling companies in the area of player protection and social responsibility in gambling.

The remaining authors declare that the research was conducted in the absence of any commercial or financial relationships that could be construed as a potential conflict of interest.

## Publisher’s note

All claims expressed in this article are solely those of the authors and do not necessarily represent those of their affiliated organizations, or those of the publisher, the editors and the reviewers. Any product that may be evaluated in this article, or claim that may be made by its manufacturer, is not guaranteed or endorsed by the publisher.
