# Supplementary material for: Gender differences and left-behind experiences in the relationship between gaming disorder, rumination and sleep quality among a sample of Chinese university students during the late stage of the COVID-19 pandemic
Source: Front Psychiatry. 2023 May 5;14:1108016. doi: 10.3389/fpsyt.2023.1108016 (PMC10196354; doi:10.3389/fpsyt.2023.1108016)
Supplement: Supplementary file 1 [file Data_Sheet_1.pdf]

Supplementary materials

Appendix S1. Frequencies of the GDT

Participants reported the GDT	N	%	Males	Females
"Never" on all items of GD	632	33.8	251	381
"Rarely" or "sometimes" for one item	1174	62.7	636	538
One indicator of GD (i.e., "often" or "very often")	39	2.1	26	13
Two indicators of GD	14	0.7	9	5
Three indicators of GD	3	0.2	2	1
all four indicators of GD	10	0.5	6	4

Appendix S2. Frequency of seven factors of sleep quality

Score	SSQ (%)	SL(%)	SD(%)	HSE (%)	SDD (%)	USM (%)	DD (%)
0	509 (27.2)	825 (44.1)	554 (29.6)	1520 (81.2)	446 (23.8)	1827 (97.6)	598 (31.9)
1	1087 (58.1)	722 (38.6)	1247 (66.6)	229 (12.2)	1272 (67.9)	26 (1.4)	626 (33.4)
2	244 (13.0)	277 (14.8)	50 (2.7)	51 (12.7)	148 (7.9)	15 (0.8)	480 (25.6)
3	32 (1.7)	48 (2.6)	21 (1.1)	72 (3.8)	6 (0.3)	4 (0.2)	168 (9.0)

Note: SSQ = Subjective sleep quality, SL = Sleep latency, SD = Sleep duration, HSE = Habitual sleep efficiency, SDD = Sleep disturbance, USM = Used sleep medication, DD = Daytime dysfunction.

Appendix S3. Edge weight matrix of the domain-level network among total sample

Variable	gd1	gd2	gd3	gd4	RE	PQSI
gd1	0.000	0.389	0.162	0.106	0.017	0.017
gd2	0.389	0.000	0.356	0.208	0.024	0.024
gd3	0.162	0.356	0.000	**0.476**	0.095	0.095
gd4	0.106	0.208	0.476	0.000	0.004	0.004
RE	0.044	0.038	0.000	0.039	0.000	0.203
PQSI	0.017	0.024	0.095	0.004	**0.203**	0.000

Appendix S4. Centrality measures per variable of the domain-level network among total sample

Variable	Betweenness	Closeness	Strength	Expected influence
gd1	-0.878	-0.366	-0.009	-0.009
gd2	0.627	0.726	0.901	0.901
gd3	**1.379**	**1.327**	**1.128**	**1.128**
gd4	-0.878	0.355	0.344	0.344
RE	-0.878	-1.450	-1.211	-1.211

PQSI	0.627	-0.592	-1.153	-1.153

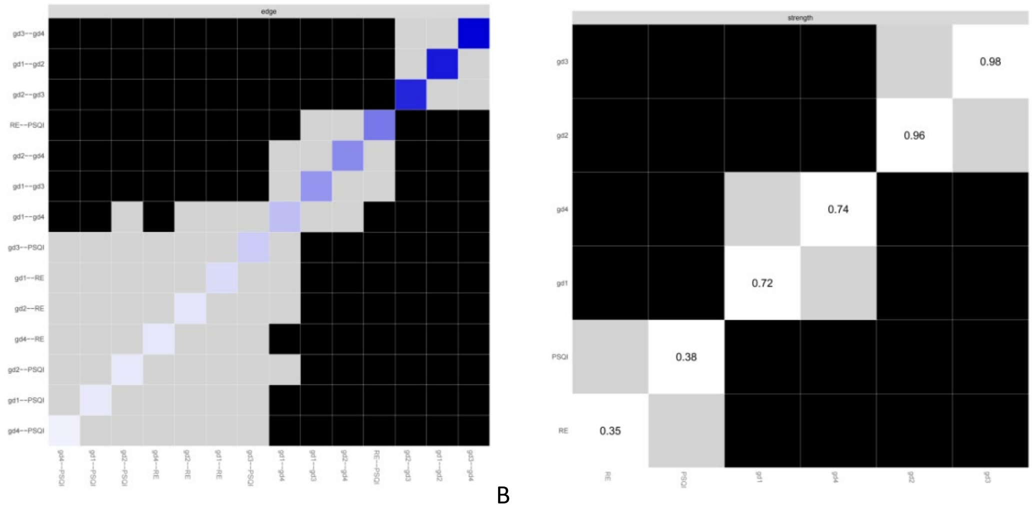

B C

Appendix S5. (A) Bootstrapped difference tests (α = 0.05) between edge-weights that were non-zero in the estimated network and (B) node strength of the multivariables. Gray boxes indicate nodes or edges that do not differ significantly from one-another and black boxes represent nodes or edges that do differ significantly from oneanother. Colored boxes in the edge-weight plot correspond to the color of the edge, and white boxes in the centrality plot show the value of node strength.

Appendix S6. Edge weight matrix of the item-level network among total sample

Variable	r1	r2	r3	r4	r5	r6	r7	r8	r9	r10	gd1	gd2	gd3	gd4	SSQ	SL	SD	HSE	SDD	USM	DD
r1	0.000	0.130	0.351	0.053	0.072	0.085	0.000	0.000	0.000	0.000	0.036	0.000	0.000	0.030	0.000	0.000	0.021	0.000	0.000	0.000	0.000
r2	0.130	0.000	0.239	0.210	0.050	0.000	0.000	-0.017	0.385	0.000	0.000	0.000	0.000	0.025	-0.002	-0.005	0.000	-0.009	0.000	0.073	0.010
r3	**0.351**	0.239	0.000	0.424	-0.018	0.039	0.099	0.079	0.000	0.000	0.000	0.001	0.000	0.000	0.000	0.000	0.020	0.000	0.000	-0.086	0.045
r4	0.053	0.210	**0.424**	0.000	0.000	0.004	0.098	0.000	0.028	0.156	0.000	0.024	0.000	0.000	0.000	0.000	0.000	0.000	6.104e-4	0.037	0.000
r5	0.072	0.050	-0.018	0.000	0.000	0.123	0.062	0.000	0.048	0.225	-0.007	0.000	-0.010	0.027	0.000	0.000	0.000	0.000	-0.010	-0.064	-0.109
r6	0.085	0.000	0.039	0.004	0.123	0.000	0.167	0.284	0.000	0.083	0.000	-0.029	0.000	-0.079	-0.017	0.000	-0.014	0.000	0.000	-0.028	0.027
r7	0.000	0.000	0.099	0.098	0.062	0.167	0.000	0.401	0.141	0.000	0.000	0.000	-0.004	0.000	0.000	0.000	0.000	0.000	0.000	0.060	0.009
r8	0.000	-0.017	0.079	0.000	0.000	0.284	**0.401**	0.000	0.083	0.139	0.024	0.000	-0.003	-0.030	0.000	0.000	0.000	0.011	0.009	0.029	0.028
r9	0.000	**0.385**	0.000	0.028	0.048	0.000	0.141	0.083	0.000	0.280	0.000	0.000	0.000	0.055	0.000	0.000	0.000	0.000	0.000	0.098	0.000
r10	0.000	0.000	0.000	0.156	0.225	0.083	0.000	0.139	0.280	0.000	0.000	0.033	2.268e-4	0.000	0.000	0.004	0.000	0.000	0.080	-0.076	0.034
gd1	0.036	0.000	0.000	0.000	-0.007	0.000	0.000	0.024	0.000	0.000	0.000	0.379	0.166	0.109	0.000	0.008	0.000	0.000	0.000	-0.037	0.013
gd2	0.000	0.000	0.001	0.024	0.000	-0.029	0.000	0.000	0.000	0.033	**0.379**	0.000	0.350	0.202	0.000	0.000	0.000	0.000	0.012	0.000	0.008
gd3	0.000	0.000	0.000	0.000	-0.010	0.000	-0.004	-0.003	0.000	2.268e-4	0.166	**0.350**	0.000	0.455	0.046	0.000	0.000	-0.002	0.000	0.016	0.056
gd4	0.030	0.025	0.000	0.000	0.027	-0.079	0.000	-0.030	0.055	0.000	0.109	0.202	**0.455**	0.000	0.031	-0.007	0.000	0.000	0.000	0.075	-0.027
SSQ	0.000	-0.002	0.000	0.000	0.000	-0.017	0.000	0.000	0.000	0.000	0.000	0.000	0.046	0.031	0.000	0.342	0.000	0.084	0.199	0.069	0.250
SL	0.000	-0.005	0.000	0.000	0.000	0.000	0.000	0.000	0.000	0.004	0.008	0.000	0.000	-0.007	**0.342**	0.000	-0.027	0.079	0.223	0.194	0.038
SD	0.021	0.000	0.020	0.000	0.000	-0.014	0.000	0.000	0.000	0.000	0.000	0.000	0.000	0.000	0.000	-0.027	0.000	0.436	0.000	0.086	0.179
HSE	0.000	-0.009	0.000	0.000	0.000	0.000	0.000	0.011	0.000	0.000	0.000	0.000	-0.002	0.000	0.084	0.079	**0.436**	0.000	0.000	0.012	-0.106
SDD	0.000	0.000	0.000	6.104e-4	-0.010	0.000	0.000	0.009	0.000	0.080	0.000	0.012	0.000	0.000	0.199	0.223	0.000	0.000	0.000	0.092	0.285
USM	0.000	0.073	-0.086	0.037	-0.064	-0.028	0.060	0.029	0.098	-0.076	-0.037	0.000	0.016	0.075	0.069	0.194	0.086	0.012	0.092	0.000	0.000
DD	0.000	0.010	0.045	0.000	-0.109	0.027	0.009	0.028	0.000	0.034	0.013	0.008	0.056	-0.027	0.250	0.038	0.179	-0.106	0.285	0.000	0.000

Appendix S7. Centrality measures per variable of the item-level network among total sample

Variable	Betweenness	Closeness	Strength	Expected influence
r1	-0.991	-0.399	-1.415	-0.390
r2	-0.707	0.475	0.789	1.034
r3	0.333	0.209	**2.221**	**1.495**
r4	-0.802	-0.083	0.090	0.779
r5	0.333	1.173	-1.132	-2.142
r6	0.806	0.439	-0.228	-0.984
r7	-0.423	-0.011	0.124	0.769
r8	-0.991	0.183	0.675	0.783
r9	0.901	1.528	0.572	1.154
r10	0.239	1.317	0.530	0.433
gd1	-1.275	-2.094	-1.404	-0.775
gd2	0.239	-1.533	0.105	0.526
gd3	-0.234	-0.844	0.517	0.930
gd4	1.847	-0.567	0.778	0.018
SSQ	-0.802	-0.099	0.120	0.635
SL	-0.234	0.269	-0.529	-0.057
SD	0.333	-0.923	-1.384	-0.741
HSE	-1.275	-1.529	-1.640	-1.621
SDD	-0.896	0.270	-0.639	0.127
USM	2.036	1.511	0.656	-1.417
DD	1.563	0.705	**1.196**	-0.556

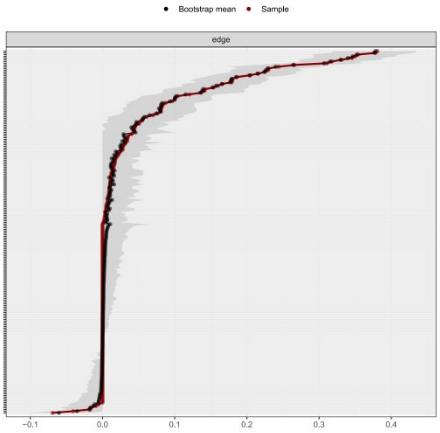

A

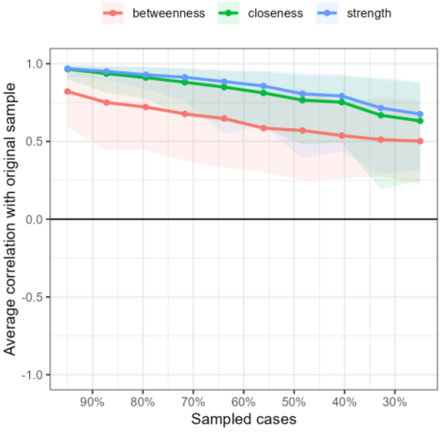

B

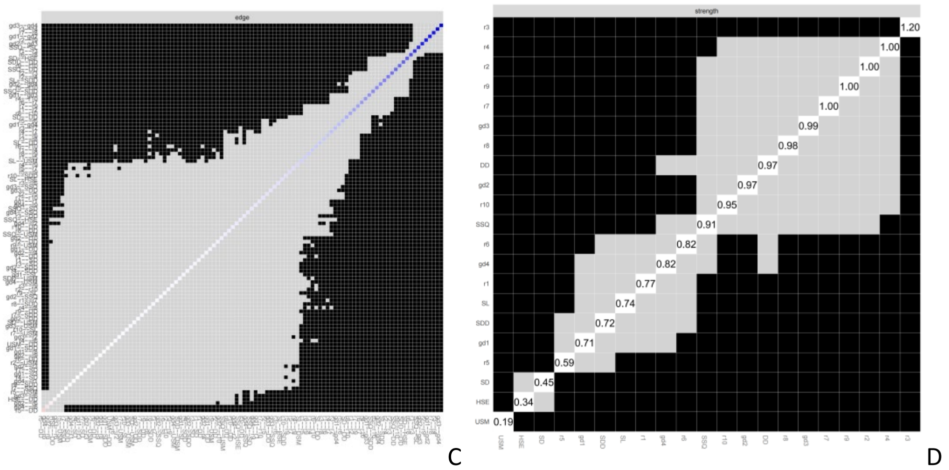

Appendix S8. Bootstrapped confidence intervals of estimated edge-weights (A) and Case-dropping bootstrap procedure for node strength (B), (C) Bootstrapped difference tests (α = 0.05) between edge-weights that were non-zero in the estimated network and (D) node strength of the multivariables.

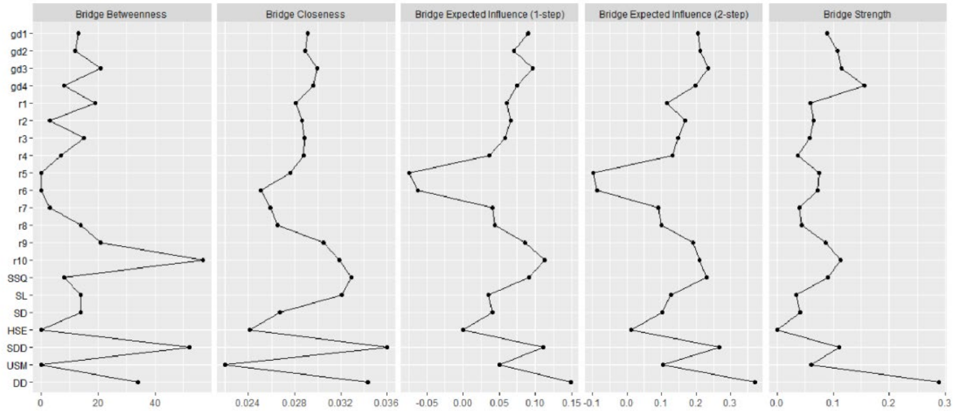

Appendix S9. Bridge centrality estimates for each node in the network. Bridge centrality including Betweenness, Closeness, Expected Influence (1-step), Expected Influence (2-step), and Strength in the item-level network.

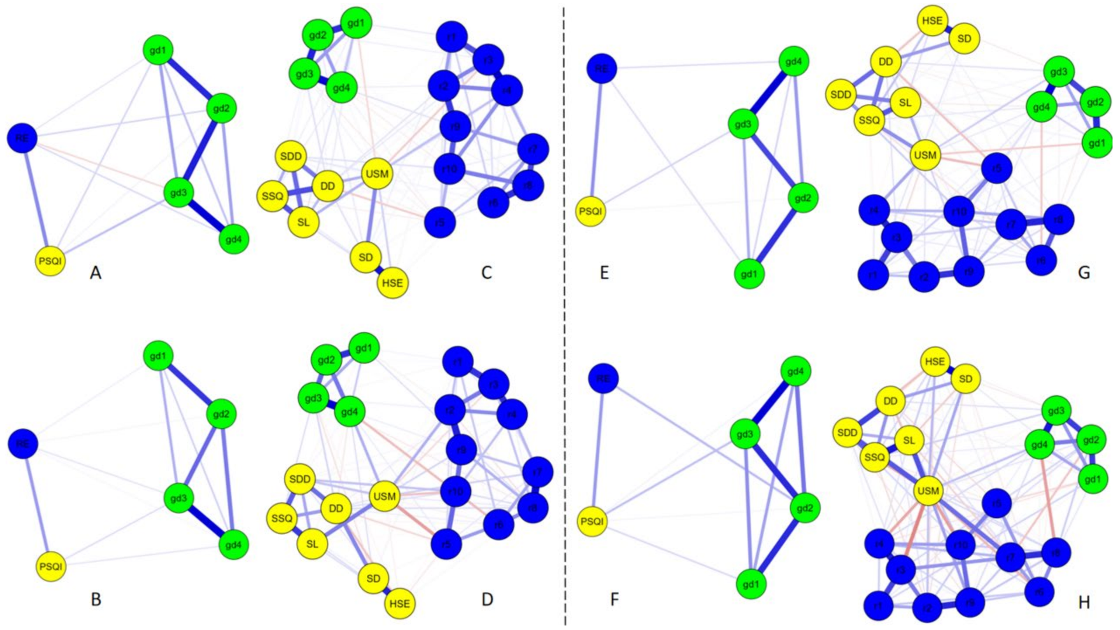

Appendix S10. EBICglasso model based on the domain-level (A, B, E, & F) and the item-level (C, D, G & H) network analysis according to the relationships between GD, rumination and sleep quality between gender and different left-behind experiences (Males = A, C; Females = B, D; Non-left-behind experience = E, G; Left-behind experience = F, H). Note Note: gd1 ~ gd4 = Gaming disorder; RE, r1 ~ r10 = Rumination; PQSI = Sleep quality, SSQ = Subjective sleep quality, SL = Sleep latency, SD = Sleep duration, HSE = Habitual sleep efficiency, SDD = Sleep disturbance, USM = Used sleep medication, DD = Daytime dysfunction.

Appendix S11. Edge weight matrix of the domain-level network between gender

Variable	Males						Females					
	gd1	gd2	gd3	gd4	RE	PQSI	gd1	gd2	gd3	gd4	RE	PQSI
gd1	0.000	0.382	0.167	0.106	0.046	0.072	0.000	0.393	0.154	0.087	0.035	0.000
gd2	0.382	0.000	0.401	0.166	0.071	0.023	0.393	0.000	0.294	0.258	0.019	0.002
gd3	0.167	0.401	0.000	**0.454**	-0.068	0.111	0.154	0.294	0.000	**0.498**	0.052	0.064
gd4	0.106	0.166	0.454	0.000	0.064	0.000	0.087	0.258	0.498	0.000	0.017	0.051
RE	0.046	0.071	-0.068	0.064	0.000	0.208	0.035	0.019	0.052	0.017	0.000	0.196
PSQI	0.072	0.023	0.111	0.000	0.208	0.000	0.000	0.002	0.064	0.051	0.196	0.000

Appendix S12. Centrality measures per variable of the domain-level network between gender

Variables	Males				Females			
	Betweenness	Closeness	Strength	Expected influence	Betweenness	Closeness	Strength	Expected influence
gd1	-0.761	-0.337	-0.021	0.125	-0.627	-0.180	-0.114	-0.114
gd2	0.952	1.008	0.845	0.994	0.878	0.669	0.787	0.787
gd3	**1.523**	**1.177**	**1.355**	**1.065**	**1.630**	**1.126**	**1.078**	**1.078**

gd4	-0.761	0.184	0.030	0.176	-0.627	0.696	0.622	0.622
RE	-0.761	-1.456	-1.035	-1.331	-0.627	-1.380	-1.178	-1.178
PSQI	-0.190	-0.576	-1.173	-1.030	-0.627	-0.931	-1.195	-1.195

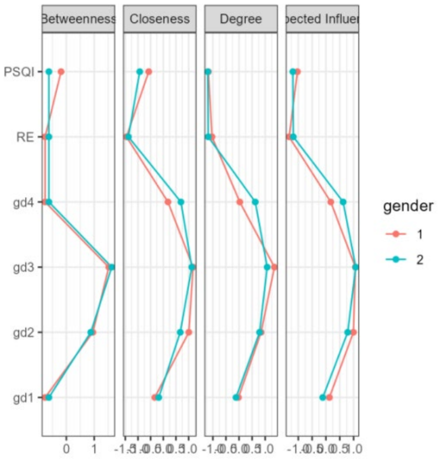

Appendix S13. Standardized estimates of node centrality in the domain-level network between males (1) and females (2).

Appendix S14. Centrality measures per variable of the item-level network between gender

Variables	Males				Females			
	Betweenness	Closeness	Strength	Expected influence	Betweenness	Closeness	Strength	Expected influence
gd1	-0.194	-1.449	-0.484	-0.712	-1.122	-1.583	-1.564	-0.778
gd2	-0.253	-1.535	0.459	0.716	0.026	-0.806	-0.543	0.389
gd3	0.338	-1.382	1.048	0.857	-0.649	-0.105	0.829	0.648
gd4	0.101	-1.360	-0.319	-0.367	1.511	0.475	**1.400**	0.535
r1	-1.022	0.262	-1.044	-0.529	-1.055	-0.963	-1.516	-0.423
r2	-0.253	0.968	0.731	0.836	1.376	0.825	1.365	1.228
r3	-0.135	0.985	**2.566**	**1.711**	-0.244	-0.310	1.229	1.310
r4	1.934	1.595	1.024	1.258	-0.987	-0.293	-0.348	0.486
r5	-0.490	-0.151	-1.400	-2.430	-0.582	1.028	-0.605	-1.986
r6	-1.022	-0.806	-0.631	-0.600	-0.244	0.467	-0.190	-0.892
r7	-0.845	0.045	0.284	0.634	-0.785	-0.064	-0.006	0.793
r8	-0.017	-0.076	0.337	0.458	-0.717	0.300	1.087	0.972
r9	0.811	1.144	0.784	1.055	0.363	0.789	0.131	0.957
r10	0.870	1.226	0.115	0.492	0.701	0.924	0.659	0.470
SSQ	-0.313	0.109	0.369	0.384	-0.649	0.148	-0.117	0.507

SL	-0.549	-0.117	-0.228	0.203	-0.042	0.840	-0.965	-0.339
SD	0.101	-0.087	-1.105	-0.551	0.161	-1.494	-1.357	-0.819
HSE	-1.022	-0.779	-1.938	-1.524	-1.122	-2.054	-1.432	-1.935
SDD	-1.022	-0.441	-0.820	-0.516	0.228	-0.070	0.040	0.691
USM	2.999	1.709	-0.030	-1.116	2.524	2.176	**1.206**	-1.425
DD	-0.017	0.139	0.280	-0.257	1.309	-0.230	0.697	-0.391

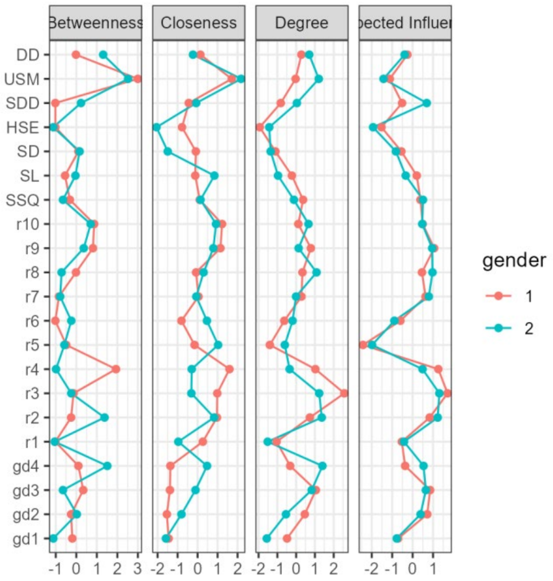

Appendix S15. Standardized estimates of node centrality in the item-level network between males (1) and females (2).

Appendix S16. Edge weight matrix of the domain-level network between Left-behind experiences

Variable	No						Yes					
	gd1	gd2	gd3	gd4	RE	PQSI	gd1	gd2	gd3	gd4	RE	PQSI
gd1	0.000	0.402	0.158	0.080	0.068	0.000	0.000	0.354	0.185	0.153	0.000	0.063
gd2	0.402	0.000	0.355	0.197	0.000	0.020	0.354	0.000	0.354	0.230	0.111	0.020
gd3	0.158	0.355	0.000	**0.492**	0.000	0.081	0.185	0.354	0.000	**0.424**	0.000	0.102
gd4	0.080	0.197	0.492	0.000	0.068	0.000	0.153	0.230	0.424	0.000	0.000	0.022
RE	0.068	0.000	0.000	0.068	0.000	0.210	0.000	0.111	0.000	0.000	0.000	0.166
PSQI	0.000	0.020	0.081	0.000	0.210	0.000	0.063	0.020	0.102	0.022	0.166	0.000

Appendix S17. Centrality measures per variable of the domain-level network between Left-behind experiences

Variables	No				Yes			
	Betweenness	Closeness	Strength	Expected influence	Betweenness	Closeness	Strength	Expected influence

gd1	-0.339	0.130	-0.006	-0.006	-0.645	-0.222	0.080	0.080
gd2	0.678	0.688	0.818	0.818	**1.291**	**1.167**	**1.010**	1.010
gd3	**1.695**	**1.152**	**1.166**	1.166	1.291	1.069	0.998	0.998
gd4	-0.339	0.459	0.394	0.394	-0.645	0.169	0.298	0.298
RE	-0.848	-1.315	-1.130	-1.130	-0.645	-0.959	-1.335	-1.335
PSQI	-0.848	-1.114	-1.241	-1.241	-0.645	-1.224	-1.052	-1.052

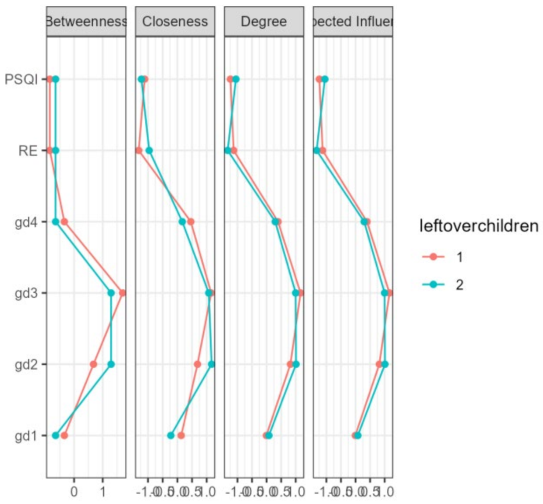

Appendix S18. Standardized estimates of node centrality in the domain-level network between non-left-behind experience (1) and left-behind experience (2).

Appendix S19. Centrality measures per variable of the item-level network between left-behind experiences

Variables	No				Yes			
	Betweenness	Closeness	Strength	Expected influence	Betweenness	Closeness	Strength	Expected influence
gd1	-0.311	-1.071	-1.007	-0.767	-0.444	-1.588	-0.830	-0.648
gd2	-0.687	-1.385	-0.080	0.369	-0.541	-1.174	0.006	0.932
gd3	0.317	-0.732	0.990	0.907	-0.493	-1.078	-0.234	0.747
gd4	-0.311	-1.041	0.722	-0.008	0.999	-0.485	-0.099	-0.077
r1	-1.000	-0.296	-1.296	-0.332	-0.444	-0.152	-1.412	-0.485
r2	-0.499	0.321	0.518	0.976	-0.444	-0.511	0.241	0.608
r3	-0.122	0.600	**1.739**	1.426	0.518	1.103	1.008	1.549
r4	1.508	1.378	0.741	0.986	-0.396	0.146	0.282	0.604
r5	0.191	1.164	-0.779	-2.065	-0.493	-0.755	-1.297	-2.155
r6	-0.122	-0.139	-0.170	-1.098	-0.396	-0.038	-0.502	-0.847

r7	-0.875	-0.476	-0.176	0.609	0.181	1.651	0.485	1.074
r8	0.317	0.032	0.854	1.009	-0.060	0.827	-0.017	0.218
r9	-0.624	0.236	0.121	0.831	-0.011	0.172	0.124	1.796
r10	0.756	0.956	0.248	0.773	-0.011	0.307	-0.234	-0.198
SSQ	0.944	0.914	1.236	0.944	-0.493	-0.070	0.205	0.029
SL	-1.000	-0.019	-0.955	-0.316	-0.011	0.488	0.061	0.214
SD	-0.436	-1.338	-1.909	-0.687	-0.541	-0.569	-0.459	-1.024
HSE	-1.000	-1.644	-1.309	-1.651	-0.589	-1.102	-0.711	-1.415
SDD	-0.750	0.012	-1.068	-0.178	0.181	0.655	0.160	0.716
USM	3.139	2.204	0.406	-1.377	3.982	2.588	**3.596**	-0.788
DD	0.567	0.322	1.175	-0.352	-0.493	-0.413	-0.372	-0.850

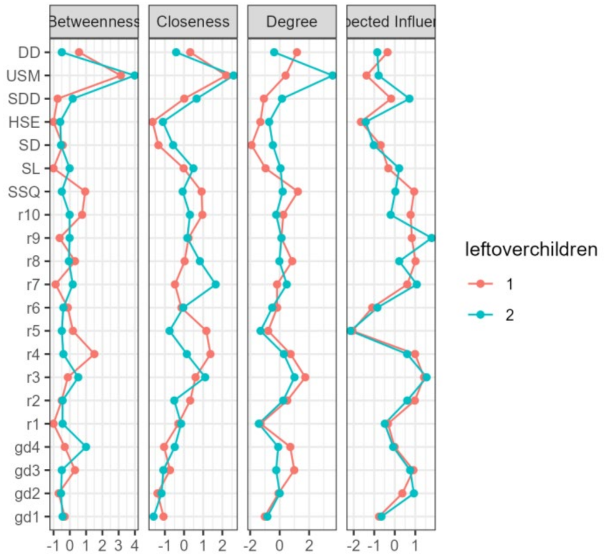

Appendix S20. Standardized estimates of node centrality in the item-level network between non-left-behind experience (1) and left-behind experience (2).
